# Plantar Verrucous Carcinoma Misdiagnosed as a Recalcitrant Wart: Diagnostic Pitfalls and Destructive Local Progression

**DOI:** 10.7759/cureus.106660

**Published:** 2026-04-08

**Authors:** Circe K Ruiz Palafox, Patricia Flores Troche, Irisdey Espinoza Urzua, Ximena Gintare Alvarez Estrada, Regina Mejía Vázquez

**Affiliations:** 1 School of Medicine, Benemérita Universidad Autónoma de Puebla, Puebla, MEX; 2 Department of Internal Medicine, Hospital General Regional 200 Tecámac, Tecámac, MEX; 3 Department of Internal Medicine, Hospital General Dr. Darío Fernández Fierro, Mexico City, MEX; 4 School of Medicine, Instituto Politécnico Nacional, Mexico City, MEX; 5 Department of Internal Medicine, Hospital General de Zona 194, Instituto Mexicano del Seguro Social, Mexico City, MEX

**Keywords:** carcinoma cuniculatum, cutaneous squamous cell carcinoma, deep biopsy, diagnostic delay, immunotherapy, pembrolizumab, plantar verrucous carcinoma, recalcitrant wart

## Abstract

Chronic plantar verrucous lesions represent a frequent diagnostic challenge, as they often mimic benign conditions such as plantar warts. However, the continued assumption of a benign diagnosis despite lack of therapeutic response reflects not only delayed biopsy, but also insufficient consideration of relevant differential diagnoses, including cutaneous malignancies and deep infections. Among these, verrucous carcinoma, particularly its plantar variant, carcinoma cuniculatum, is a rare but locally aggressive subtype of cutaneous squamous cell carcinoma characterized by slow growth, low metastatic potential, and progressive local destruction.

We report the case of a 76-year-old male with an 8-year history of a verrucous lesion on the right foot, demonstrating progressive enlargement and initially managed as an infectious and inflammatory condition with multiple topical and systemic therapies, without clinical improvement. Over time, the lesion evolved into a large exophytic mass with ulceration, malodorous discharge, and significant functional limitation. Imaging studies revealed suspicious inguinal lymphadenopathy without confirmed distant metastasis. A deep incisional biopsy established the diagnosis of well-differentiated squamous cell carcinoma, verrucous variant (carcinoma cuniculatum). Due to extensive locoregional involvement and a high risk of functional morbidity, the tumor was deemed initially unresectable.

Systemic treatment with pembrolizumab (200 mg every three weeks) was initiated in the setting of locally advanced unresectable disease. After six cycles, the patient demonstrated a partial clinical and radiological response, with sustained disease control and good tolerance after eleven cycles, allowing reassessment for potential surgical management.

This case illustrates how prolonged diagnostic delay, in this instance, nearly eight years, can lead to locally advanced disease, limiting initial surgical options. It underscores the importance of early biopsy and comprehensive diagnostic reassessment in chronic verrucous lesions, as well as the potential role of immunotherapy as a therapeutic alternative in selected patients with advanced verrucous carcinoma.

## Introduction

Chronic verrucous lesions of the foot represent a frequent diagnostic challenge for clinicians, as they often share morphological features with common benign entities such as plantar warts, calluses, and mechanical hyperkeratosis. Although most correspond to non-malignant conditions, a clinically relevant subset may represent cutaneous neoplasms that mimic benign lesions, leading to diagnostic delays, repeated empirical treatments, and progressive local disease [1,2].

Cutaneous squamous cell carcinoma (cSCC) is the second most common skin cancer worldwide and encompasses a broad spectrum of histopathological variants. Among these, verrucous carcinoma is an uncommon, well-differentiated subtype characterized by slow growth, a predominantly exophytic pattern, locally invasive behavior, and low metastatic potential. Despite its relatively indolent histologic appearance, its clinical significance lies in its ability to cause substantial local destruction when diagnosis is delayed [3].

The palmoplantar variant, known as carcinoma cuniculatum, was originally described by Aird in 1954 [4]. Since then, it has been recognized as an uncommon but clinically significant neoplasm, particularly due to its tendency to be misdiagnosed for years as a recalcitrant plantar wart [5,6]. This initial clinical resemblance often results in prolonged use of topical therapies, keratolytics, antibiotics, or repeated local procedures before a malignant etiology is considered and an appropriate biopsy is performed.

From a clinical standpoint, carcinoma cuniculatum may begin as an apparently trivial hyperkeratotic lesion and progress insidiously into a large exophytic verrucous mass with a broad base, deep sinus tracts, drainage of keratinous material, malodorous discharge, ulceration, and progressive induration [3]. Warning signs that should raise clinical suspicion include lesion persistence, progressive growth, lack of response to conventional treatment, malodor, pain, ulceration, and functional impairment.

The differential diagnosis of chronic verrucous lesions of the foot is broad and includes not only recalcitrant plantar warts but also deep infections, implantation mycoses, adnexal neoplasms, and conventional squamous cell carcinoma. Therefore, the diagnostic challenge lies not only in distinguishing benign from malignant lesions but also in appropriately integrating clinical context, disease evolution, and therapeutic response.

A critical factor contributing to diagnostic delay is the failure to perform a biopsy in persistent or treatment-refractory verrucous lesions, rather than the use of superficial biopsies per se. The lack of diagnostic reassessment in the setting of treatment failure significantly contributes to disease progression. When performed, biopsy should be deep and representative, as superficial samples may underestimate the infiltrative nature of the lesion [3-5].

Although its metastatic potential is low, delayed diagnosis may result in extensive local invasion, involvement of deep soft tissues, and even bone infiltration, leading to significant functional morbidity and, in advanced cases, the need for radical surgery or amputation [6]. In this context, early recognition and timely performance of a deep biopsy are essential to prevent destructive progression and preserve conservative treatment options.

We present the case of a patient with long-standing plantar verrucous carcinoma initially managed as a benign and infectious lesion, which progressed to a locally advanced stage. This case illustrates the consequences of inadequate diagnostic reassessment in chronic verrucous lesions and underscores the importance of considering malignant etiologies in persistent plantar lesions.

## Case presentation

A 76-year-old male patient with a history of chronic tobacco use presented with a verrucous lesion on the right foot, which had first appeared approximately 8 years prior to his current evaluation and had shown slow, progressive growth.

Approximately six months before referral to oncology, the lesion exhibited a change in clinical behavior, characterized by an increase in size, pain, malodor, discharge, and impaired ambulation.

Initial medical evaluations were performed several months before the definitive diagnosis. During this period, the patient received multiple treatments targeting an infectious or inflammatory process, including topical and systemic antibiotics (topical and oral clindamycin and oral dicloxacillin) as well as topical corticosteroids (hydrocortisone), administered over several weeks without clinical improvement. The lack of therapeutic response, together with continued clinical progression, defined the recalcitrant nature of the lesion.

Initial imaging evaluation with computed tomography revealed no evidence of distant pleuropulmonary disease; however, radiologically suspicious inguinal lymphadenopathy was identified. No lymph node biopsy was performed; therefore, nodal involvement was considered a radiologic suspicion rather than confirmed disease. Magnetic resonance imaging was not performed, limiting the assessment of deep tissue invasion, including fascia, tendons, and bone.

A deep incisional biopsy of the lesion was subsequently performed, selecting a representative area with greater induration and clinical features suggestive of infiltration, given the large tumor size and suspicion of malignancy. This approach allowed adequate tissue sampling while avoiding an extensive initial resection. 

The specimen consisted of a multifragmented sample measuring approximately 2.7 × 1.5 × 0.7 cm. Histopathological examination demonstrated a well-differentiated squamous neoplasm consistent with verrucous squamous cell carcinoma (carcinoma cuniculatum). Microscopically, there was proliferation of highly differentiated squamous keratinocytes with minimal cytologic atypia and low mitotic activity. The tumor exhibited a predominantly exophytic growth pattern, with marked hyperkeratosis, papillomatosis, and broad epidermal ridges extending into the dermis in a pushing pattern of invasion, characteristic of this variant.

No perineural or lymphovascular invasion was identified. Tumor extension was documented at least to the reticular dermis. Surgical margins were not evaluable, as this was an incisional biopsy rather than a complete excision. The pathological stage was reported as pT1.

The fragmented nature of the specimen limited full assessment of tumor architecture and precise determination of invasion depth. This limitation is relevant for staging and treatment planning; however, histopathological findings were sufficient to establish a definitive diagnosis. The tumor was reported as a well-differentiated squamous cell carcinoma, verrucous variant (carcinoma cuniculatum), likely associated with human papillomavirus infection.

**Figure 1:**
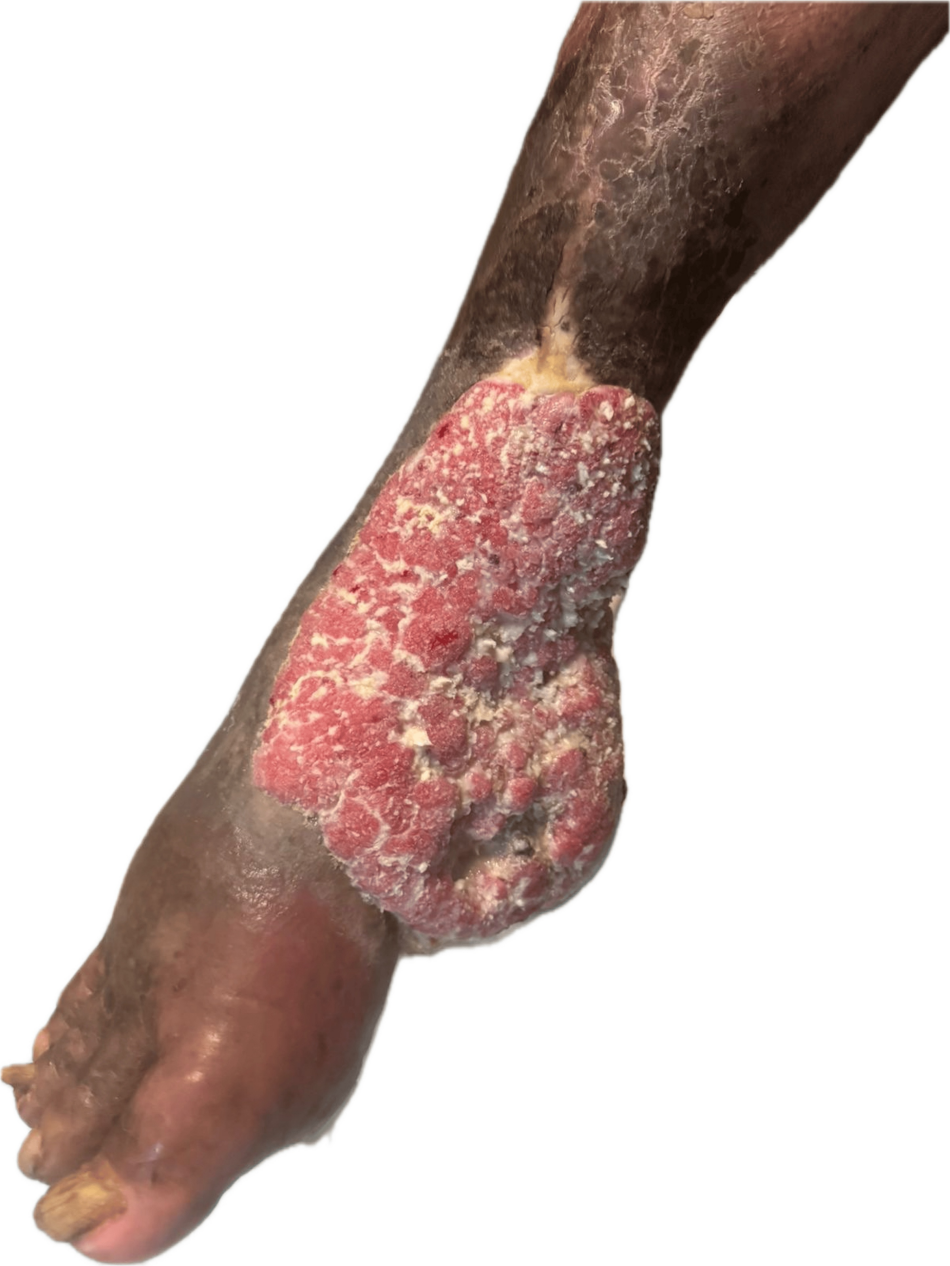
Clinical presentation of the lesion Dermatosis localized to the right foot, predominantly involving the plantar and lateral aspects with extension toward the ankle. A large exophytic, nodular mass measuring approximately 15 × 10 cm is observed, characterized by a verrucous, hyperkeratotic surface with abundant keratinous debris. The lesion exhibits well-defined borders, areas of ulceration, and multiple sinus tracts draining keratinous material.

On physical examination, a dermatosis localized to the right foot was observed, predominantly involving the dorsal and medial regions with extension to the plantar surface. The lesion consisted of an approximately 15 × 10 cm exophytic, nodular mass with well-defined borders, a verrucous and hyperkeratotic surface, a salmon-colored appearance, and multiple sinus tracts draining keratinous material, along with areas of ulceration and induration (Figure 1).

**Figure 2:**
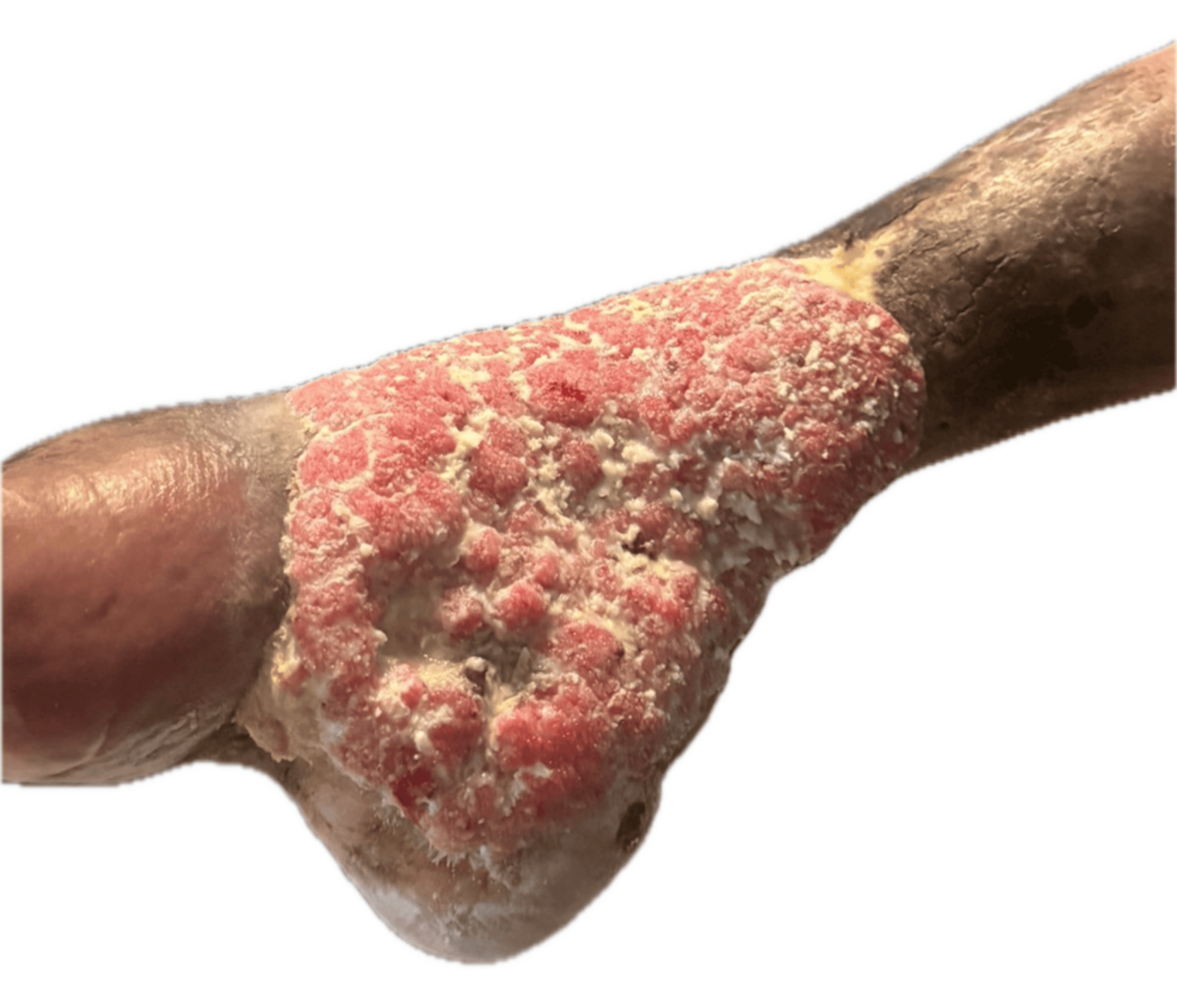
Additional clinical view of the lesion An additional view demonstrating a markedly hyperkeratotic, papillomatous exophytic tumor with well-defined borders, abundant keratinous debris, and an irregular surface architecture.

Additional clinical views highlighted the markedly hyperkeratotic and papillomatous surface, with accumulation of keratinous debris and a well-defined exophytic growth pattern (Figure 2).

Based on clinical, histopathological, and imaging findings, a diagnosis of locally advanced cutaneous squamous cell carcinoma, verrucous variant, was established.

The case was evaluated by surgical oncology; however, the patient was not considered a candidate for initial conservative surgical resection due to the large tumor size, locoregional extent, and high risk of functional morbidity, including the potential need for amputation.

Given the initial unresectability, systemic treatment with immunotherapy was initiated using pembrolizumab at a dose of 200 mg intravenously every 21 days, in the context of locally advanced disease not amenable to curative surgery or radiotherapy.

The patient received a total of 11 treatment cycles. After six cycles, a reduction in tumor size and improvement in local symptoms were observed. Imaging evaluation demonstrated a partial response according to RECIST criteria, with persistence of inguinal lymphadenopathy [7].

During treatment, the patient did not develop immune-related adverse events and showed good overall tolerance. At the most recent follow-up, the patient remained alive, clinically stable, and under active treatment, with sustained partial response. A follow-up imaging study was planned to reassess the possibility of surgical management. A chronological summary of the clinical course is presented in Table 1.

**Table 1:** Chronological clinical course and management of a patient with plantar verrucous carcinoma (carcinoma cuniculatum) RECIST: Response Evaluation Criteria in Solid Tumors

Period	Clinical event
~8 years before diagnosis	Onset of a verrucous lesion on the right foot with slow, progressive growth
~6 months before oncologic evaluation	Change in clinical behavior, including increased size, pain, fetid odor, secretion, and gait limitation
Months prior to diagnosis	Multiple treatments without response, including topical and systemic antibiotics (clindamycin, dicloxacillin) and topical corticosteroids
Initial imaging evaluation	Computed tomography showed no pleuropulmonary disease and revealed radiologically suspicious inguinal lymphadenopathy
Diagnostic stage	Incisional biopsy confirming well-differentiated squamous cell carcinoma, verrucous variant (carcinoma cuniculatum)
Oncologic assessment	Determination of initial unresectability due to extensive locoregional involvement and high surgical morbidity risk
Treatment initiation	Pembrolizumab 200 mg administered intravenously every 21 days
After 6 cycles	Clinical reduction in tumor size with partial response according to RECIST criteria [7]
Latest follow-up	Patient alive, clinically stable, and under active treatment with sustained partial response

## Discussion

The plantar verrucous carcinoma or carcinoma cuniculatum is an uncommon and well-differentiated variant of cutaneous squamous cell carcinoma that, despite its low metastatic potential, can cause significant local destruction when diagnosis is delayed [3-5]. Its importance lies not only in its rarity but also in the frequency with which it may be underestimated as a benign lesion, particularly as a recalcitrant plantar wart, which favors prolonged diagnostic delays and local tumor progression [5,6]. Diagnostic delay in carcinoma cuniculatum is common, with multiple reports documenting years of evolution under misdiagnoses, which increases morbidity and limits conservative therapeutic options.

In the present case, the prolonged clinical course, close to eight years, is notable, as well as the persistence of treatments directed toward infectious or inflammatory etiologies without therapeutic response. This pattern reflects one of the main problems in the approach to chronic verrucous lesions: the persistence of an initial diagnostic hypothesis despite the lack of improvement. More than an isolated error, this phenomenon represents a failure in clinical reassessment, which leads to diagnostic delay and disease progression.

From a therapeutic standpoint, the treatment of choice for localized verrucous carcinoma is complete surgical resection. Wide excision has traditionally been the standard approach; however, Mohs micrographic surgery is considered in many centers as the treatment of choice in selected lesions, due to its ability to evaluate 100% of surgical margins and maximize tissue preservation, which is particularly relevant in functional areas such as the foot [8]. Compared with conventional wide excision, Mohs surgery may reduce recurrence rates and optimize functional outcomes. Nevertheless, in extensive tumors or those with suspected deep invasion, the possibility of a conservative approach may be limited, and radical procedures, including amputation in advanced cases, may be required. In this regard, invasion into deep structures, including soft tissues and bone, has been described, leading to greater morbidity and therapeutic complexity [6,8,9].

In this patient, tumor extent, locoregional involvement, and the risk of significant functional morbidity led to the patient being considered unsuitable for initial conservative surgical resection. The presence of inguinal lymphadenopathy was considered a radiological suspicion without histological confirmation, avoiding the assumption of definitive metastatic disease. Likewise, magnetic resonance imaging and formal evaluation of bone invasion were not available, which limited complete staging. These limitations must be explicitly acknowledged, as they influence case interpretation and therapeutic decision-making.

In this scenario of locally advanced and initially unresectable disease, systemic treatment with pembrolizumab, a PD-1 inhibitor, was initiated. Although verrucous carcinoma has a particular biological behavior, the use of immunotherapy in this case was based on evidence extrapolated from advanced cutaneous squamous cell carcinoma, such as that reported in the KEYNOTE-629 study [10]. The documented partial response, together with clinical improvement and adequate treatment tolerance, supports the role of immunotherapy as a viable therapeutic alternative in selected patients.

From a clinical perspective, one of the most relevant aspects is the differential diagnosis of chronic verrucous lesions of the foot. These lesions should not be interpreted as a homogeneous category, but rather as a spectrum that includes benign, infectious, and neoplastic entities. A plantar wart represents the main differential diagnosis; however, it usually follows a relatively stable course and does not present progressive infiltration or significant functional impairment. In contrast, cutaneous squamous cell carcinoma should be suspected when there is progressive growth, induration, ulceration, pain, or therapeutic failure.

Chromoblastomycosis constitutes a particularly relevant differential diagnosis, as it may present as a chronic, exophytic, and deforming verrucous lesion, with great clinical similarity to keratinocytic neoplasms [11]. Likewise, cutaneous tuberculosis may manifest as verrucous or ulcerated plaques with an indolent course, acting as a clinical simulator [12]. These entities reinforce the need not to assume a single diagnosis without histological confirmation [13].

In this context, biopsy should not be considered a last resort, but rather a central diagnostic tool. The key clinical message is that any chronic verrucous lesion that is progressive or refractory to treatment should be biopsied in a timely and deep manner.

Regarding histopathological diagnosis, verrucous carcinoma is characterized by proliferation of well-differentiated keratinocytes, with minimal cytological atypia, prominent keratinization, and a predominantly exophytic growth pattern with pushing borders. In the present case, the biopsy was incisional and allowed the diagnosis to be established; however, the sample was fragmented, which prevented a complete evaluation of the depth of invasion.

This limitation is particularly relevant, as tumor depth is a key element for staging and therapeutic planning in cutaneous squamous cell carcinoma. In addition, the absence of histopathological microphotographs limits independent clinicopathological correlation, which represents a weakness of the report. Although ideally it would have been desirable to have representative histological images or a more extensive biopsy, the pathological diagnosis was conclusive and consistent with the clinical presentation and patient evolution.

From a practical perspective, this case reinforces that any chronic verrucous lesion of the foot with progressive growth, associated symptoms, or lack of therapeutic response should be considered suspicious for malignancy until proven otherwise, and requires deep biopsy as part of the initial approach, avoiding diagnostic delays that lead to locally advanced disease.

Limitations

Among the main limitations are the absence of histological confirmation of inguinal lymphadenopathy, the lack of imaging studies aimed at evaluating deep invasion, such as magnetic resonance imaging, and the inability to precisely determine tumor depth due to sample fragmentation. Likewise, the absence of histopathological microphotographs limits complete morphological correlation. These limitations restrict precise oncological staging and should be considered when interpreting the findings of the present case.

## Conclusions

Plantar verrucous carcinoma remains a diagnostic challenge due to its relatively benign clinical and histological appearance, which favors diagnostic errors and prolonged therapeutic delays. The present case demonstrates that a chronic, progressive, or treatment-refractory verrucous lesion should not be presumed benign, but rather requires a timely biopsy as part of the standard diagnostic approach in order to prevent tumor progression and limit the associated morbidity. In this case, an eight-year diagnostic delay resulted in locally advanced disease, precluding initial surgical management.

In locally advanced and initially unresectable disease, immunotherapy with PD-1 inhibitors, such as pembrolizumab, represents a viable therapeutic alternative. Its use is supported by evidence about advanced cutaneous squamous cell carcinomas, and in this case, allowed tumor control and therapeutic reassessment. Overall, this case highlights the importance of an active and stepwise diagnostic strategy in chronic verrucous lesions, as well as the need to integrate systemic treatment options in advanced scenarios to optimize clinical outcomes.
